# Early technology review during prototype development and at proof of concept: the case for developing a sequential versus a single-stage approach to early evidence development for health technologies

**DOI:** 10.1017/S0266462326103596

**Published:** 2026-04-22

**Authors:** Leslie Levin, Robert S. McDonough, Martin Cheatle, Haytham Kaafarani, Serge Korjian, Richard E. Kuntz

**Affiliations:** 1 EXCITE International, Canada; 2 Aetna Inc, USA; 3 Psychiatry, Anesthiology, Critical Care, University of Pennsylvania Perelman School of Medicine, USA; 4 Surgery, Massachusetts General Hospital, Harvard Medical School, USA; 5 Cardiology, Massachusetts General Hospital, Harvard Medical School, USA; 6 Cardiology, Baim Institute for Clinical Research Inc, USA; 7 None, USA

**Keywords:** technical assessments, investigational therapies, device approval, diffusion of innovation, evidence-based medicine

## Abstract

The effect of Early Technology Review (ETR) through early engagement with multiple stakeholders on strategic development for technologies at prototype development and proof of concept was examined through two generic case studies of relevant outcomes. In both examples, advice to companies could have significantly changed strategic direction to become more relevant to payers and clinical experts. In one instance, the advice was followed and resulted in an expedited first-in-human study and was considered for a second ETR to inform the proof-of-concept study. In the second example, it was reported that changes in strategic direction were being considered.

These reports provide descriptive accounts of very early applications of the ETR process that now spans the entire preclinical trajectory. Had the second case study at proof of concept been able to benefit from this approach at the point of prototype development, it could have avoided the costs and research through earlier advice. This begs the question whether a sequential iterative approach to evidentiary multiple stakeholder advice across the technology life cycle may reduce risk and cost while benefitting from efficiencies of applying adaptive design.

## Background

Health technologies require regulatory clearance to allow companies to market their products. Unfortunately, this has become the major focus of health technology companies for evidence development, resulting in the current lack of insights reflecting expectations of payers and clinical experts early in the development of health technologies. In the United States, which is the focus of this article, insights into expectations beyond those of the Food and Drug Administration (FDA) for regulatory clearance have been described (1). Briefly, these broader expectations only become known following regulatory clearance, which presents a problem since evidentiary requirements often differ from those of regulators. While the FDA is focused on safety and evidence that the technology performs according to the manufacturer’s claims, payers and clinicians have different priorities. Payers and clinicians are focused on comparative effectiveness analysis, clinical utility, and durability of effect, in a relevant target population against an appropriate comparator using a clinical trial design that provides quality data on agreed-upon relevant outcomes.

In Europe, early scientific advice is offered to health technology companies to help generate evidence for future technology appraisals essential for adoption of the technology by the health system, two examples of which appear in the Commentary section.

While this approach is reported to have been successful, it is mainly applied to pharmaceutical products (2). Furthermore, the absence of companies, especially from the scoping process, has come under criticism (3).

Recognizing the dissociation between regulatory and coverage processes, the FDA is facilitating early exposure by companies to non-FDA parties through its voluntary Total Product Life Cycle Advisory (TAP) Program (4), to expedite the development of “breakthrough” health technology devices that offer more effective treatment or diagnosis for life-threatening or irreversibly debilitating conditions. The TAP process is designed to provide early, proactive, and solution-oriented engagement to assist companies to “better understand potential challenges related to adoption of and patient access to their innovative devices” (4). This is currently restricted to ophthalmic, cardiovascular, neurologic, physical medicine, orthopedic, and radiological health-related devices.

Failure to address stakeholder expectations early in the development and evaluation of health technologies could account for the low level of acceptance by the Centers for Medicare and Medicaid (CMS) for coverage determination subsequent to FDA clearance for breakthrough health technology devices (5). This results in a precarious pathway to reimbursement and uncertainty in clinician support to change practice and/or develop treatment guidelines. EXCITE International, a nonprofit organization, was founded to address this reality by offering inclusive direct early engagement by health technology companies with payers and clinical experts through its Early Technology Review (ETR) (1) designed to create a premarket awareness of these stakeholder expectations.

The ETR, a fee-based advisory process, is a comprehensive evidence-based process conducted through six 2-hr panel meetings over 4 months (1). The process is supported by an early evidence review and based on an a priori agreed-upon framework of expectations developed by EXCITE, the company, and an EXCITE-appointed expert panel consisting of US payers, clinical thought leaders, and clinical trial methodologists. The panels are unique to each technology reviewed, and while payers are drawn from the standing Payers Advisory Committee (PAC), clinical thought leaders are appointed according to their expertise in the clinical area related to the technology in question. Proceedings remain confidential to panelists, but the final report becomes the intellectual property of the company, which may release its contents at its discretion.

The ETR has demonstrated that meeting payer, clinical expert, and clinical trial methodologist expectations is dependent on good quality evidence, selection of the appropriate target population, clinical outcomes – including durability of response – and comparator(s), and evidence of economic benefit if the technology has outcomes similar or superior to comparators. Many of these determinants form the basis for comparative effectiveness analysis, which is important to payers and clinical experts.

The ETR also explores opportunities and obstacles to adoption, considered important for companies in their approach to technology development and evaluation. This experience has demonstrated congruence of expectations of these expanded stakeholders and is important to payers in coverage determination, according to EXCITE’s PAC. The Payers Advisory Committee has developed a confidential, comprehensive guidance document related to engagement with payers based on published information and ETR experience to date. This guidance document allows companies to understand the coverage process and forms a consistent basis for discussions with payers on the panel.

Attempts are made to address potential conflicts of interest relating to the ETR process. Clinical experts are not encumbered by the company, their involvement being through EXCITE, which in turn does not publicly endorse technologies undergoing the ETR. Furthermore, panelists cannot currently receive reimbursement from the company for any reason, and noncompany representatives cannot have shares in the company. While the company is invited to designate up to three panelists, they serve the purpose of providing technical perspectives relating to the health technology and are excluded from any discussion leading to advice provided by the panel. However, being on the panel allows the company to engage with panelists for their insights into product development. In the event that the panel chair calls for a vote regarding advice, company representatives are excluded from the process.

Views expressed by payers on the panel are those of each senior policy decision-maker, do not necessarily reflect the views of their host insurance companies, and are not intended for use by payers in their coverage determinations.

Any engagement between the company and the panel, while not being encouraged, can only occur through EXCITE.

## Case studies examining the ETR undertaken early and very early in the technology life cycle

While earlier consideration of stakeholder expectations provides important information for companies developing health technologies (1), it is not known how early in the lifecycle these perspectives should be sought.

This article explores the ETR approach undertaken very early during prototype development and further downstream, but still early in the life cycle, closer to pivotal trial development. Two generalized case studies from completed ETRs (1) are presented to demonstrate the impact stakeholder involvement can have at these stages of the early life cycle. Case studies presented were selected to demonstrate how the ETR provides early insights into payers and clinical experts that prepare the company for downstream coverage determination and clinical adoption. In the interests of conciseness and protection of confidentiality and protection of proprietary issues, only highlighted outcomes are presented and extracted from detailed and comprehensive reviews. The companies associated with each of the technologies provided permission to publish these outcomes in an anonymized format.

While each of the two case studies examines health technologies at separate and distinct phases of the life cycle and differ fundamentally in their intended use, there are similarities in what is expected from the ETR process, which apply to both case studies, including the following:

Satisfying payers and clinicians to reimburse and change clinical practice, respectively, requires high-quality evidence that the new intervention is safer and/or more effective than existing alternatives or, if equivalent, is of economic benefit.

The number of primary outcomes should be kept to a minimum unless a gated approach (6) is used, since increased outcomes require increasing the sample size to maintain power. Secondary outcomes are only considered if the primary outcome has been met.

Outcomes change according to the stage of development of the technology. For example, outcomes such as progression-free survival and durability of effect can only be addressed in the later stages of technology development.

Since subjective outcomes are considered less important in decision-making, the companies are urged, wherever possible, to focus on objective outcomes.

### A priori framework of expectations for the ETR

A summarized a priori framework of expectations applicable to both case studies that guided the expert panel’s discussions and was agreed to by the panel, the company, and EXCITE appears below:

- Review of preclinical and/or clinical effectiveness and safety considerations of the technology and of likely alternatives through an early evidence review, panel input, and FDA expectations, if available.
- Discussion on the potential value-add of the technology compared to other physical and biologically modified targeted and systemic therapies.
- High-level decision determinants that could be important to payers and clinicians.
- Likely complications/unintended consequences associated with the technology, compared to existing alternative approaches.
- Evidence that might be required for use in humans.
- High-level advice on study designs that could satisfy expectations of payers and clinicians.
- Potential barriers or opportunities to adoption of the technology, if found to be effective.

## Case study #1: ETR undertaken to inform prototype development

For colorectal cancers in which resection is not possible, a colostomy is required, and the surgical procedure results in significant morbidity. The objective of the photothermic technology reviewed at the phase of prototype development was to reduce tumor burden as a colostomy-sparing procedure or by allowing an end-to-end anastomosis.

The photothermic approach uses gold nanoparticles (GNRs), which vibrate to produce tumoricidal temperatures in the presence of near-infra-red (NIR) light at an appropriate wavelength. This was followed by in vitro confirmation of the tumoricidal effect while protecting normal cells. Cancer cells can be killed at a temperature of 45 C whereas normal cells remain viable up to 55 C. Enhanced tumor cell kill was demonstrated in mice, in addition to interleukin2-enhanced remote cell kill attributed to cell-mediated immunity following antigen exposure from intratumor cell death.

Protype development involved intratumor injection or systemically administered GNRs with preferential intratumor sequestration due to an enhanced permeability and retention effect, followed by exposure to a NIR light source able to penetrate up to 1–2 cm of tissue.

### Panel advice

The panel advised that intratumor injection of GNRs was preferable to the systemically delivered alternative, since the dose could be more accurately determined with less likelihood of extra-tumoral activation by the NIR light source.

Furthermore, the intended selection of distal colorectal cancer posed potential technical difficulties given that the NIR beam for tumors required >1–2 cm penetration. Deeper penetration would also make it difficult to control the dose and treatment of the regional lymph nodes. Stability of the light source placed in the rectum was a concern due to uncontrolled peristaltic movement. Decreased precision in monitoring thermal distribution in a nonvisualized tumor would add difficulty and pose safety hazards. An NIR light source for use in humans is under development and will include calibration ports and temperature monitoring capabilities, as well as allowing a broader beam to illuminate a wider area while requiring longer exposure rates.

The panel questioned whether a safe temperature can be accurately maintained for a colorectal tumor. While this can be addressed using a focused light source, and dose/volume algorithms were being developed to inform duration of dosing in the clinical setting, it would be preferable to select a tumor site in which control of the light source can be instantaneously modified according to accurate thermal dose distribution measurements with reduced tissue toxicity.

The panel recommended it would be safer to use unresectable melanoma as the initial model. The extracorporeal light source would be stable, manipulated with ease, and the intratumor thermal distribution would be easier to measure. Most importantly, local tissue toxicity would not be as serious as it would be for colorectal cancer, in which penetration of the colon or rectal wall or affecting anal sphincter control were serious adverse events to be considered. Unresectable melanoma would provide a better benefit–risk ratio, while improving the feasibility of successfully concluding a study.

The panel encouraged the further development of the NIR light source for use in humans. The unresected melanoma model provided the best opportunity to apply the technology to its maximum potential while minimizing risk to the patient.

Locally advanced breast cancer was another model considered, though several established treatment options, including brachytherapy, are already available compared to unresected melanoma, which is resistant to most treatments, apart from recent reports of benefit using immunotherapy. This technology could therefore meet an unmet need.

The panel pointed out that whatever model was selected, it would be unlikely that the results would be generalizable to other cancers, and separate studies would be required for each additional tumor site. They further emphasized the importance of developing techniques to inject tumors with an even distribution of the GNRs.

The panel commented that activation of GNRs could release materials associated with local and/or systemic toxicity. However, biocompatibility and lack of toxins have been investigated, especially when nanorods are coated with polyethylene glycol. A distribution and elimination study is being considered. The panel did not involve itself in other safety issues since it regarded safety considerations as being under the purview of the FDA.

### Selection of a comparator(s)

Payers and clinicians are focused on comparative effectiveness against an accepted alternative (comparator) technology, if available. The panel suggested that the selection of treatment-resistant unresectable melanoma might be more straightforward in this regard, since there are unlikely to be established effective comparators apart from immunotherapy.

### Clinical trial perspectives

The company considered reengaging with the ETR process at the proof-of-concept phase following first-in-human studies, and most likely to coincide with their pre-submission to the FDA. This would represent an iterative ETR that reflects the progressive nature of payer and clinical expert advice guided by new data.

If unresectable melanoma were to be selected as the model, progression-free survival would be a preferred outcome to overall survival, since the latter may not be feasible, given the likelihood of a long follow-up period.

In addition to outcomes suggested by the ETR panel and the FDA, it was suggested that the company explore outcomes that were used for approval of drugs cleared by the FDA and the National Comprehensive Cancer Network for unresectable melanoma, noting that the latter are often used by payers as a basis for coverage determinations.

### Additional factors relating to downstream coverage determination and adoption

The company will need to confirm comprehensive Good Laboratory Practice safety, access to GNRs, an NIR light device for use in humans, and confirmatory in vivo studies on local and systemic immune modulation if it makes the latter claim.

A payer pointed out that further downstream, the company will need to consider the basis for funding by payers through factors such as charging a facility or administration fee, intended use as a separate device, or bundled as part of a diagnostic-related group.

### Follow-up and overall comments on case study #1

The company pivoted towards using unresectable melanoma as the tumor model to explore the clinical utility of its technology. This alternative target population would be more likely to be acceptable to clinicians and payers and could demonstrate a value-add to existing treatment, while being safer for patients through an improved benefit–risk ratio when compared to colorectal cancer. Positioning the NIR source without an invasive procedure would reduce a barrier to adoption. If effective, additional tumor sites could be considered in the future.

The company was able to undertake first-in-human studies within 12 months of the ETR and is poised to undertake a proof-of-concept study, informed by data from the first-in-human study.

## Case study #2: ETR undertaken as part of a proof-of-concept study

The clinical diagnosis of Alzheimer’s disease (AD) is based on cognitive examination, routine laboratory tests, and neuroimaging. An accurate diagnosis can take years (7), and a diagnosis based on clinical criteria is reached 70–80 percent of the time (8). A definitive diagnosis can only be made by histological analysis of human brain samples confined to autopsy studies. Technologies that can confirm or exclude AD-related pathology in living people are therefore needed.

Amyloid-PET is an accurate diagnostic imaging tool for AD (9;10). With the advent of drugs to treat AD based on monoclonal antibodies to amyloid, the demand for Amyloid-PET scanning has increased, since it is required for a decision whether to offer this expensive treatment. However, accessibility and cost are concerning.

This ETR was requested for a fundus hyperspectral camera designed to scan the retina and, through AI, determine characteristics unique to AD by differentiating between normal and pathological amyloid status. It was reported to provide early, accurate diagnosis, by bypassing the limitations of the blood–brain barrier for blood tests, and is presumed to closely relate to cerebral amyloid. The technology was intended for use by primary care physicians in deciding whether to refer patients to a specialist especially given the increased advent of new drugs. The intended use case was the detection of phenotypic changes in the retina that correlate with PET amyloid status to aid in the evaluation of AD and other causes of cognitive decline. It was designated by the FDA as a breakthrough device.

Panelists indicated that referral to a specialist is for the assessment of cognitive impairment and not only AD. Furthermore, often there are comorbid conditions contributing to cognitive impairment, and it can be difficult to ascertain which of these is dominant. This has prompted the development of a multi-analyte diagnostic panel to include non-amyloid, non-p-tau markers of neurodegeneration.

The panel pointed to emerging blood tests aimed at the detection of amyloid and p-tau, which are proving to be very accurate. While the technology was intended to be an adjunct to blood testing, payers indicated they would scrutinize a new test added to existing tests used to make a treatment decision. Under those circumstances, it would be possible to consider reimbursing blood biomarkers and the technology in question if the tests were of equal effectiveness and cost, but a case would need to be made for how they would fit into the diagnostic/screening workplan. This advice may not apply to Europe, where amyloid and p-tau blood tests were not yet available.

Given the current focus on convenient amyloid and p-tau blood tests, clinical experts advised that an alternative or additional focus for the technology would be to delineate which of the comorbid dementia conditions were contributing most to cognitive impairment, given its ability to detect retinal changes based on AI. The technology was also considered potentially important to better assess patients most likely to respond to current therapies or those with lower amyloid levels, so that quantification could become an additional focus for the further development of the technology.

From a payer’s perspective, the impact on clinical decision-making will be the measure of clinical utility, which is key to coverage determination. Funding will be guided by demonstrated complementarity as an adjunct to another test or improved outcome. Consideration for coverage may occur without direct interaction with payers, this being triggered, for example, by the designation of an appropriate Current Procedural Terminology (CPT) code, or where there is marketing pressure. CPT codes, determined by the American Medical Association, describe medical services and procedures performed by doctors and healthcare professionals, and are used by insurance companies to process claims and determine reimbursement for services. Interest by payers occurs where there is sufficient evidence of improvement over existing alternatives, or equivalence with respect to performance and cost, and indications for use are clearly stated. Using a nonspecific code for billing for services that fall outside predefined categories, such as the 99 code, may be successful for low-cost technologies. The company needs to present comparative data and a clear explanation of where the technology fits in the clinical pathway.

The target age demographic for this technology is over 65 years, which is the population served by CMS. For this reason, any clinical trial should include a large number of patients that represent this demographic. Communication channels with CMS were offered to the company to get more precise information regarding their expectations. Approaches to access the new FDA TAP and the CMS Transitional Coverage for Emerging Technologies program for FDA breakthrough-designated technologies were shared with the company.

It was pointed out that when applying for a CPT code, the company should be aware that the process has become increasingly specific to a particular technology, with fewer opportunities to apply codes based on other similar technologies. If a technical component needs to be applied, that could limit the use of the technology since the number of fundoscopic examinations is limited.

### Overall comments on ETR for case study #2

This technology was considered a potentially important addition to the diagnosis and possibly screening for dementia. While the panel advised that its role as a screening modality is likely to be overshadowed by the growing focus on amyloid and p-tau blood tests, the technology would likely be considered especially useful in providing information in the assessment of the relative contribution of comorbid types of dementia. Payers advised that if used as an adjunct to blood tests, the incremental contribution to the assessment of AD needs to be demonstrated for coverage determination. Blood tests and this technology may replace the amyloid PET scan to make a diagnosis that warrants access to a drug if accuracy can be demonstrated.

In conclusion, the company was advised to consider changing its strategic direction and to focus on differentiation between comorbid dementias and their respective contribution to dementia through quantitative analysis.

### Comment on very early engagement

The early engagement by companies with payers and clinical experts, as described in the two case studies presented, provides an opportunity for stakeholder perspectives in health technology development and early evaluation. The case studies demonstrate the utility of this approach during prototype development and at proof of concept.

The first case study showed that early evidence development that responds to expectations of payers and clinical experts is not only possible but could avoid unnecessarily complicated high-risk approaches that might, in the end, not be supported by these stakeholders. The expeditious advance to a positive first-in-human study now positions the company to proceed to a proof-of-concept study most likely to meet the expectations of payers and clinical experts.

The second study would have resulted in a change in strategic direction, but these details were not available. However, had the opportunity been available at the point of prototype development, an earlier change in strategic direction for prototype development and clinical utility would have been possible.

Key to the ETR approach and experience is the value-add of combining the perspectives of both payers and clinical experts in the process. There is synergism between these perspectives, which payers involved in the ETR process have indicated normally forms part of coverage determination. Furthermore, the early perspective of clinical experts is essential to adoption and clinical guideline development.

The demonstrated utility of the ETR process as early as prototype development begs the question whether the process should become iterative from very early in technology development, with payer and expert panels interacting with the company at each phase of the life cycle. While this would result in additional cost, it could improve the chances of a positive coverage determination and clinical adoption. Results from each phase would inform the next phase as appropriate. Given that a sequential approach could be resource-intensive for payer and clinical expert participation, selection criteria for this approach would need to be developed.

An iterative ETR process could play a key role in increasing the adoption of adaptive trial designs, particularly in areas where there is data equipoise. Adaptive trials allow for pre-planned modifications to study design or procedures based on interim data. Modifications may include changes to sample size, treatment arms, randomization ratios, or enrichment strategies without undermining the trial’s internal validity or data integrity.

Adaptive approaches are likely to be more efficient, informative, and ethical than trials with a fixed design since they often make better use of resources, might require fewer participants, and can be applied across all phases of clinical research (11;12).

The demonstrated engagement with clinical experts and payers at any discrete stage of the preclinical life cycle will continue to provide benefit to companies wishing to address multiple stakeholder expectations during early technology development.

The FDA has and continues to provide companies with guidance through the regulatory pathway leading to regulatory clearance. The ETR has, for the first time, provided an opportunity for companies to derive the same early guidance related to coverage and clinical adoption.

The ETR process is not unique. Two other examples are worth noting. The first is Early Scientific Advice provided by the National Institute for Health and Clinical Excellence (NICE) (13) in which the company submits a briefing document with specific questions. This is followed by a three-hour dialogue with experts, patients, and other agencies, and finally nonbinding feedback to refine development plans, intended to provide evidence to develop products that are more likely to succeed in the UK health system.

The second example is HI-NL, based in the Netherlands (14). Following an analysis of the relevant existing care pathways, and decision-making stakeholders or relevant reimbursement structures, a three-hour roundtable is held with the company in which stakeholders provide their perspective on what is needed to evaluate and successfully implement the health technology. Collective guidance is then provided on how to move the technology forward successfully to reimbursement and adoption, and forms the basis for an Innovation Guide shared with the company.

These early evidence initiatives have similar objectives to the ETR, though less comprehensive than the cumulative 12-hr ETR panel meetings in which there is direct engagement with multiple payers, clinical experts, and methodologists, underpinned by an early evidence review.

### Limitations

Information presented was limited by compliance with confidentiality and the need to protect proprietary information and interests as set out in agreements with the companies. While the sequential process requires further validation to determine its final impact, experience to date warrants further development of this approach.

The ETR experience to date is almost exclusively focused on United States-based payer and clinical expert expectations, and no attempt has been made to generalize this to other jurisdictions.

The ETR approach focuses on clinical outcomes and does not address broader perspectives such as patient or ethical considerations.
